# Disinfectant-Assisted Preparation of Hierarchical ZSM-5 Zeolite with Excellent Catalytic Stabilities in Propane Aromatization

**DOI:** 10.3390/nano14090802

**Published:** 2024-05-05

**Authors:** Peng Zhang, Jianguo Zhuang, Jisheng Yu, Yingjie Guan, Xuedong Zhu, Fan Yang

**Affiliations:** 1 Engineering Research Center of Large-Scale Reactor Engineering and Technology, East China University of Science & Technology, Ministry of Education, Shanghai 200237, China; zhangpengxxx123@163.com (P.Z.);; 2 State Key Laboratory of Green Chemical Engineering and Industrial Catalysis, Sinopec Shanghai Research Institute of Petrochemical Technology Co., Ltd., Shanghai 201208, China

**Keywords:** ZSM-5 (**MFI**), propane aromatization, one-step, surface modification

## Abstract

A series of quaternary ammonium or phosphonium salts were applied as zeolite growth modifiers in the synthesis of hierarchical ZSM-5 zeolite. The results showed that the use of methyltriphenylphosphonium bromide (MTBBP) could yield nano-sized hierarchical ZSM-5 zeolite with a “rice crust” morphology feature, which demonstrates a better catalytic performance than other disinfect candidates. It was confirmed that the addition of MTBBP did not cause discernable adverse effects on the microstructures or acidities of ZSM-5, but it led to the creation of abundant meso- to marco- pores as a result of aligned tiny particle aggregations. Moreover, the generation of the special morphology was believed to be a result of the coordination and competition between MTBBP and Na^+^ cations. The as-synthesized hierarchical zeolite was loaded with Zn and utilized in the propane aromatization reaction, which displayed a prolonged lifetime (1430 min vs. 290 min compared with conventional ZSM-5) and an enhanced total turnover number that is four folds of the traditional one, owing to the attenuated hydride transfer reaction and slow coking rate. This work provides a new method to alter the morphological properties of zeolites with low-cost disinfectants, which is of great potential for industrial applications.

## 1. Introduction

Light aromatics, especially BTX (benzene, toluene, and xylene) are fairly important basic raw materials that find applications in pharmacy, synthetic fibers, solvents, etc., which are usually obtained by the catalytic reforming of naphthalene [1,2,3,4]. It is of great importance to explore alternative strategies for BTX production to address concerns about crude oil depletion. On the other hand, propane is an important byproduct produced during petroleum processing and an important component of natural gas with the beneficial properties of adequacy and low cost [5,6,7]. Therefore, the aromatization of propane has emerged as a promising source of BTX, which can alleviate the severe dependence of aromatic production on petroleum resources [8,9,10,11]. 

Metal-modified zeolites have been used to enhance propane dehydrogenation and aromatic production [12,13,14], among which Zn/ZSM-5 showed superior catalytic performance in propane activation and aromatization. In addition, the Zn/ZSM-5 catalyst has been applied to industrial-scale processes such as the Topas process from Topsoe and the Alpha process from Sanyo [15]. Previous studies have revealed the bifunctional mechanism of propane aromatization over Zn/ZSM-5. Propane is firstly dehydrogenated on the Zn site or Lewis acid site (LAS) to obtain propylene and ethylene, followed by oligomerization and cyclization of intermediates on the Brønsted acid site (BAS) [16,17].

Based on the above discussion of the bifunctional mechanism, recent research studies have primarily focused on the modification of ZSM-5, which mainly involves structure optimization and acid regulation [18,19]. Specifically, structural modification can improve diffusion performance and reduce coke deposition, while multi-metal synergy can enhance the catalytic performance of a catalyst [20,21,22,23]. Furthermore, the catalytic performance of a catalyst can also be promoted by changing the ratio of BAS and LAS in the catalyst [24]. Although numerous modification methods have been proposed to enhance catalytic properties in the propane aromatization reaction, common catalysts are still not optimal with respect to their rapid coke deactivation [20,25,26].

Hierarchical zeolites or nanocrystalline zeolites can effectively shorten diffusion pathways, thereby reducing the probability of side reactions and suppressing carbon deposition, which is a well-known solution to extend catalytic lifetime [27,28,29]. Compared with its nano-sized counterpart, the approaches to obtain hierarchical ZSM-5 are versatile, and the recovery of hierarchical ZSM-5 from precursor solutions is much easier [30]. Hence, the preparation of hierarchical ZSM-5 has remained a hot field of research during the past decades. Briefly, hierarchical ZSM-5 can be obtained through “top-down”, methods like desilication, or through “bottom-up” routes with the assistance of zeolite growth modifiers (e.g., organosilane, quaternary ammonium salts, etc.) [31,32,33]. However, the post-treatment usually results in a low yield of target materials because of SiO_2_ leaching, and the zeolite growth modifiers [34,35] are very cost-sensitive in terms of industrial applications. Consequently, exploiting less expensive ways to create hierarchical zeolites is of great importance from the perspective of large-scale applications. Similar to the widely reported amphiphilic surfactant used in the synthesis of hierarchical ZSM-5, disinfectants containing tetra-ammonium salts or tetra-phosphonium salts also comprise long hydrocarbon tails and N^+^/P^+^ cation heads. Thus, these disinfectants could be potential zeolite growth modifiers or even templates to obtain hierarchical or nano-sized zeolites, with the advantage of lower prices relative to amphiphilic surfactants. Nevertheless, to the best of our knowledge, these disinfectants have only been reported in the synthesis of zeolites for unknown reasons.

In this work, a series of commonly used tetra-ammonium or tetra-phosphonium disinfectants were utilized as zeolite growth modifiers to reveal their potential effects on the final products. The results showed that disinfectants could only effectively exert their role in moderate Na^+^ environments, and a series of ZSM-5 zeolites with different morphologies could be acquired by changing the disinfectants employed. The obtained zeolites were systematically characterized and evaluated in propane aromatization, and the zeolite synthesized with the assistance of methyltriphenylphosphonium bromide, owning a “rice crust” feature, disclosed the most excellent catalytic performances. This work provides a new method for altering the morphological properties of zeolites with low-cost disinfectants, which is of great potential for industrial applications.

## 2. Experimental Section

### 2.1. Materials

Tetrapropylammonium hydroxide (TPAOH, 25 wt.% aqueous solution) and sodium meta aluminate (NaAlO_2_) were purchased from Shanghai Dibo Chemical Technology Co., Ltd. (Shanghai, China). Ethanol (AR) was purchased from Shanghai Lingfeng Chemical Reagent Co., Ltd. (Shanghai, China). Aluminum isopropoxide was purchased from TCI Co., Ltd. (Shanghai, China) Tetraethyl orthosilicate (TEOS, 99%), NaOH (98%), NH_4_Cl (99%), and Zn(NO_3_)_2_·6H_2_O (99%) were all purchased from Shanghai Titan Scientific Co., Ltd. (Shanghai, China). Methyltriphenylphosphonium bromide (MTBBP, 98%), Didecyl Dimethyl Ammonium Chloride (DDAC, 99%), Tetradecyltributylphosphonium chloride (TBTCP, 98%), and Benzyldimethyldodecylammonium chloride (DDBAC, 98%) were purchased from Shanghai Macklin Biochemical Technology Co., Ltd. (Shanghai, China).

### 2.2. Catalyst Preparation

The precursor solution for the preparation of ZSM-5 was synthesized with the composition of 30 TEOS: 1 NaAlO_2_: 2 NaOH: 900H_2_O:6 TPAOH: 3 X, where X was labeled as a different phosphorus modifier. Firstly, 6.51 g TPAOH was dissolved in 16.72 g deionized water followed by 8.33 g TEOS addition and stirring for 6 h. Then, 0.17 g NaAlO_2_ was introduced into the above solution and stirred for another 3 h. Next, different phosphorus modifiers were added (1.42 g MTBBP/1.74 g TBTCP/1.44 g DDAC/1.36 g DDBAC), and the final mixtures were stirred for 3 h again before sealing into a Teflon-lined stainless-steel autoclave to carry out hydrothermal crystallization at 170 °C for 72 h under dynamic conditions.

After hydrothermal synthesis, the product was filtrated, followed by drying at 120 °C overnight and calcinating at 550 °C for 6 h. The sample was converted into the H-form by performing three ion exchanges in 1 M NH_4_Cl solution at 80 °C for 6 h and then calcined at 550 °C for 6 h. After the ion exchanges, the sample was labeled as X-ZSM-5, while the sample synthesized without the phosphorus modifier added was labeled as C-ZSM-5.

Conventional incipient wetness impregnation was used to prepare Zn catalysts. Typically, Zn(NO_3_)_2_·6H_2_O corresponds to 2 wt.%. Zn was dissolved in deionized water and was added dropwise to X-ZSM-5. After drying overnight at 120 °C, the sample was calcined at 550 °C for 6 h. The final samples were remarked as Zn/C-ZSM-5, Zn/MTBBP-ZSM-5, Zn/TBTCP-ZSM-5, Zn/DDBAC-ZSM-5, and Zn/DDAC-ZSM-5.

### 2.3. Catalyst Characterization

Powder X-ray diffraction (XRD) was conducted on a Bruker D8-Advance (Brucker, Karlsruhe, Germany) with monochromatized Cu Kα radiation (40 kV and 80 mA).

Nitrogen (N_2_) adsorption–desorption isotherms were measured on a Micromeritics ASAP2460 analyzer (Norcross, GA, USA). The specific surface areas were calculated by the Brunauer–Emmett–Teller (BET) method, the microporous volumes and surfaces were determined by the t-plot method, and the pore size distributions were obtained from the adsorption isotherm branch by the NLDFT method.

Scanning electron microscopy (SEM) micrographs were obtained on a Zeiss Sigma500 instrument (Zeiss, Oberkochen, Germany) with an accelerating voltage of 0.02–30 kV.

An inductively coupled plasma atomic emission spectrometer (ICP-AES) was used to determine the accurate Si/Al ratios of the catalysts on Agilent 710-ES (Agilent, Santa Clara, CA, USA) [36].

The temperature-programmed desorption of ammonia (NH_3_-TPD) dates were studied by an ASAP2720 Micromeritics device (Norcross, GA, USA)fitted with a thermal conductivity detector (TCD).

Fourier transform infrared spectra (FT-IR) and pyridine adsorption infrared spectra (Py-IR) were measured on a Thermo Fisher Nicolet IS50 infrared spectrometer (Thermo Fisher Scientific, Waltham, MA, USA) with a CaF_2_ window and vacuum system at 0.482 cm^−1^ optical resolution. The determination of the infrared spectrum at the -OH vibration region was made after sample treatment under high vacuum conditions for 1 h at 500 °C. Py-IR spectral data were obtained after the adsorption and desorption of pyridine at 200, 300, and 400 °C. The Brønsted and Lewis acids were quantified according to the formulas reported by Emeis [37].

Thermogravimetric (TG) analysis was used to analyze the carbon deposits of the spent catalysts using a PerkinElmer TGA 8000 (PerkinElmer, Waltham, MA, USA) under air flow from room temperature to 800 °C.

### 2.4. Catalyst Evaluation

A fixed-bed reactor was utilized to evaluate the propane aromatization reaction at 550 °C under atmospheric pressure. A total of 0.3 g 20–40 mesh catalyst was packed in a quartz tube reactor with an inner diameter of 8 mm. The feed ratio was n(N_2_): n(C_3_H_8_) = 7:3, corresponding to a propane space velocity of 3000 h^−1^.

The gaseous products consisting mainly of C1~C3 species were analyzed online using Shimadzu’s hydrogen flame ionization detector (FID) and TCD detector (Shimadzu, Tokyo, Japan). The detectors were connected to a PLOT-Q column and TDX-01 column, respectively. The liquid phase aromatic products, including benzene (C_6_H_6_), toluene (C_7_H_8_), and ethylbenzene/p-xylene/m-xylene/o-xylene (C_8_H_10_), were measured online using Agilent’s FID detector connected to a DB-WAX column (Agilent, Santa Clara, CA, USA). Propane conversion and aromatic selectivity were calculated based on the carbon number. The conversion of propane and the selectivity of products were calculated by the carbon atom conservation method and the area normalization method. The calculation formulas are described in Equations (1) and (2) as follows:

(1) $$X_{propane}=\frac{n_{p1}-n_{p2}}{n_{p1}}\times 100\%$$

(2) $$S_{i}=\frac{n_{i}}{\sum n_{i}-n_{p2}}\times 100\%$$

where *X_propane_* is the conversion of propane, *S_i_* is the selectivity of different products, *n_p_*_1_ and *n_p_*_2_ are the molar amounts of propane before and after the reaction, respectively, and n_i_ is the molar amount of different product.

## 3. Results and Discussion

### 3.1. Catalytic Characterization

#### 3.1.1. Texture Properties

**Figure 1:**
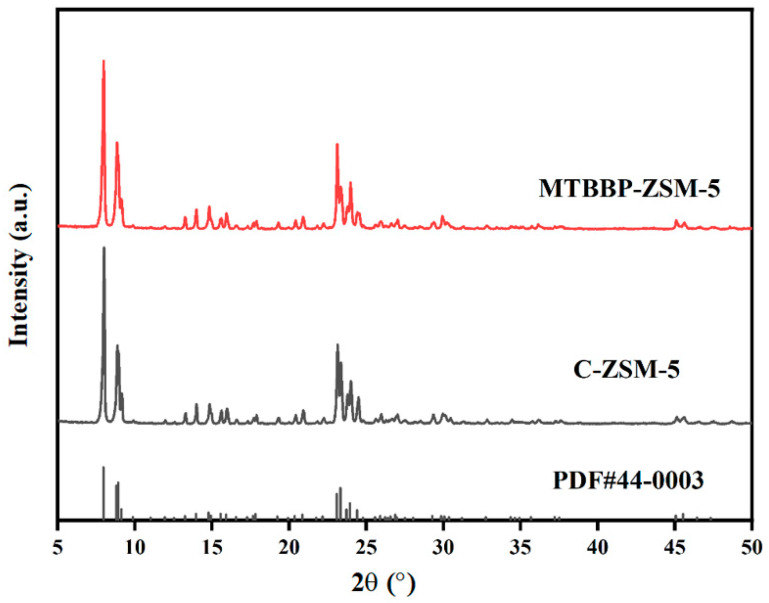
XRD patterns of MTBBP-ZSM-5 and C-ZSM-5.

MTBBP, TBTCP, DDAC, and DDBAC are all traditional disinfectants used in daily life. The ZSM-5 samples acquired with the assistance of these disinfectants were firstly subjected to a series of characterization to identify their physiochemical properties. The powder XRD patterns of C-ZSM-5 and MTBBP-ZSM-5 are shown in Figure 1. It can be seen that both profiles exhibit sharp diffraction peaks within 7.7–7.9° and 22.5–25°, which matches well with the standard patterns out of **MFI** topology. No peaks suggesting amorphous or impurities aside from the **MFI** structure could be detected, suggesting the successful formation of ZSM-5 zeolite and the total conversion of raw materials [38]. Relative crystallinities (R.C.s) were calculated from the areas underneath the peaks within 5~26°, by defining the value of C-ZSM-5 as 100%. The R.C.s were 93%, 90%, 88%, and 91% for MTBBP-ZSM-5, TBTCP-ZSM-5, DDAC-ZSM-5, and DDBAC-ZSM-5, respectively (Figure 1 and Figure S1). Actually, the catalytic performances of the samples synthesized with the addition of TBTCP, DDAC, and DDBAC were not very compelling; hence, their properties will not be discussed in detail. Obviously, only slight differences in relative crystallinities could be found, suggesting that the addition of disinfectants did not cause adverse effects for these final products.

**Figure 2:**
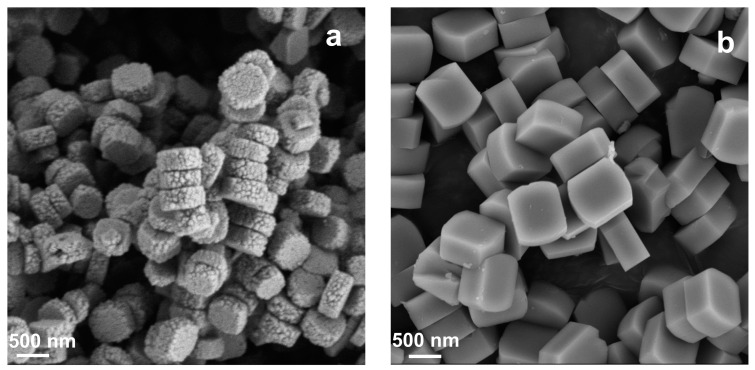
SEM micrographs of MTBBP-ZSM-5 (**a**) and C-ZSM-5 (**b**).

**Figure 3:**
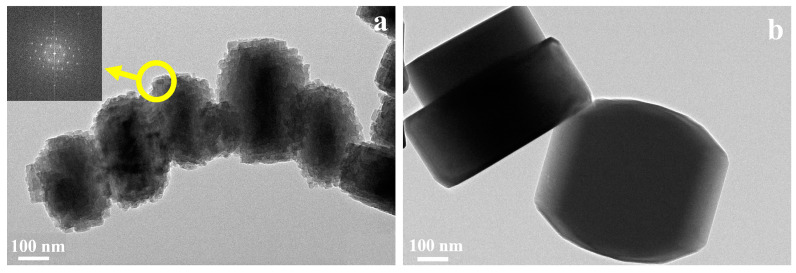
TEM micrographs of MTBBP-ZSM-5 (**a**) and C-ZSM-5 (**b**).

The SEM micrographs of synthesized samples are presented in Figure 2. C-ZSM-5 prepared in conventional hydrothermal conditions displayed uniform hexagonal particles of ca. 500 nm, with clear contours, smooth surfaces, and rigid corners (Figure 2b). Similarly, MTBBP-ZSM-5 was also composed of ~500 nm hexagonal particles; however, different from C-ZSM-5, a “rice crust” feature could be observed for the particles therein, which has a much coarser surface composed of tiny secondary crystals (Figure 2a). The SEM analyses of ZSM-5 samples synthesized utilizing other disinfectants can be found in Figure S2, which also illustrates relatively coarser surfaces but with rather ununiform size distributions, indicating their weak interactions with zeolites. Therefore, it can be concluded that MTBBP plays a significant role in the morphology of ZSM-5. A further discussion of possible growth mechanisms will be made in the following sections of this paper. The TEM pictures of C-ZSM-5 and MTBBP-ZSM-5, as illustrated in Figure 3, further corroborated that C-ZSM-5 was constructed by separated hexagonal crystals with easily recognized margins, while the elementary units of MTBBP-ZSM-5 were surrounded by tiny particles attached to the surfaces. Interestingly, the selected area electron diffraction patterns, as shown in Figure 3a, manifested a spot-like pattern, hinting at an oriented attachment during zeolite growth.

**Figure 4:**
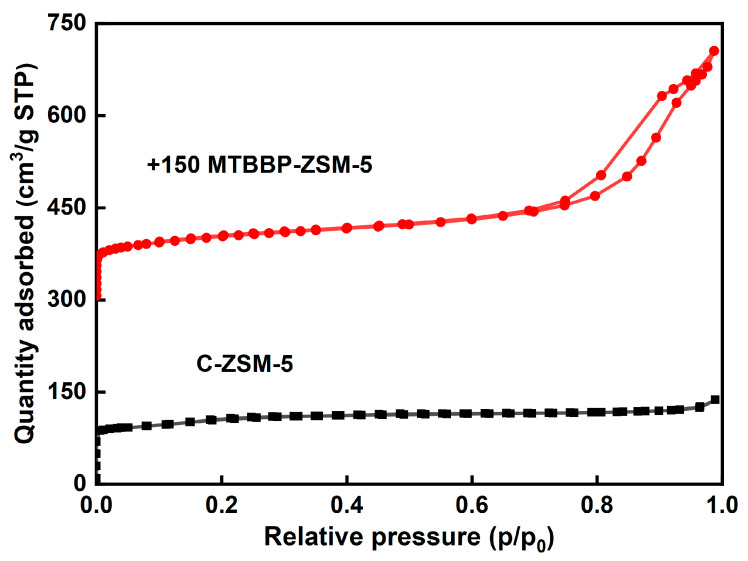
N_2_ adsorption–desorption isotherms of different samples.

**Figure 5:**
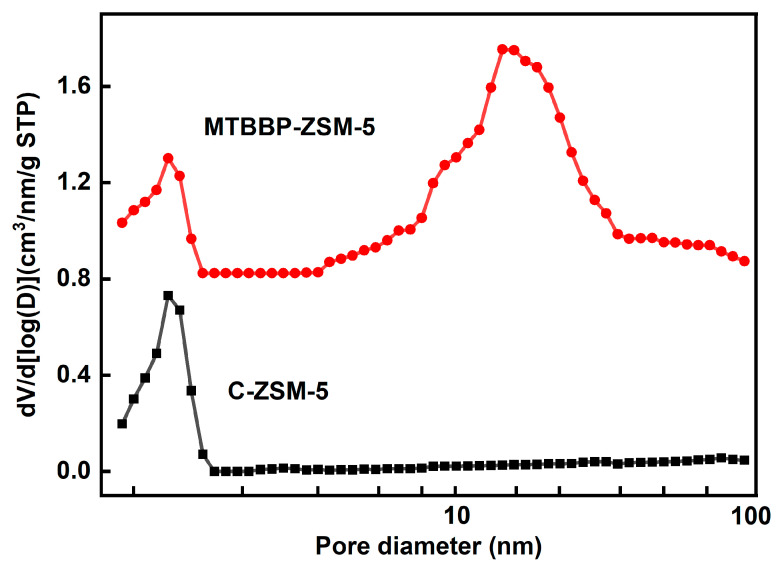
Pore size distributions of different samples.

The pore and surface information was analyzed by N_2_ adsorption–desorption. The isotherms and related structure parameters of the synthesized samples are shown in Figure 4, Figure 5, Table 1 and Table S1, respectively. MTBBP-ZSM-5 showed a type Ⅳ isotherm and C-ZSM-5 showed a type Ⅰ isotherm, according to the IUPAC classification [39]. A steep increase at relative pressure p/p_0_ <0.01 was found in both isotherms, which was ascribed to the rapid absorption of N_2_ within the micropores. As for C-ZSM-5, the adsorption soon reached a plateau at p/p_0_ = 0.2, and the uptake at high p/p_0_ regions was not discernable, indicating that C-ZSM-5 is a microporous material. In contrast, a hysteresis loop and an uptake were found at p/p_0_ > 0.9 in the isotherms of MTBBP-ZSM-5, which proves the existence of macropores in MTBBP-ZSM-5.

The exact values of the pore and surface parameters are summarized in Table 1. Both samples possess similar micropore surfaces (~180 m^2^·g^−1^) as well as micropore volumes (0.11 cm^3^·g^−1^), consistent with their intrinsic **MFI** topology. Nevertheless, the total specific surface of MTBBP-ZSM-5 (373 m^2^·g^−1^) was significantly higher than that of C-ZSM-5 (322 m^2^·g^−1^), which resulted from a larger external specific surface (195 m^2^·g^−1^ vs. 141 m^2^·g^−1^). Another evident feature was that the mesopore volume of MTBBP-ZSM-5 (0.29 cm^3^·g^−1^) was larger than C-ZSM-5 (0.12 cm^3^·g^−1^). It is possible that the differences in pore volumes and specific surfaces were ascribed to the coarser surfaces. The pore size distribution (PSD) curves of C-ZSM-5 and MTBBP-ZSM-5 derived from the adsorption branch using the NLDFT method are compared in Figure 5. The PSD curve of C-ZSM-5 shows a unique peak at 0.56 nm due to the intrinsic micropores of **MFI** topology [40] and a flat region within 5~100 nm. In addition to the sharp peak at 0.56 nm, another peak centering at 20 nm as well as a shoulder at 70 nm can also be observed for MTBBP-ZSM-5, indicating the existence of meso- to marco-pores. Hence, MTBBP-ZSM-5 can be regarded as a hierarchical zeolite [41,42,43].

#### 3.1.2. Acidic Properties

**Figure 6:**
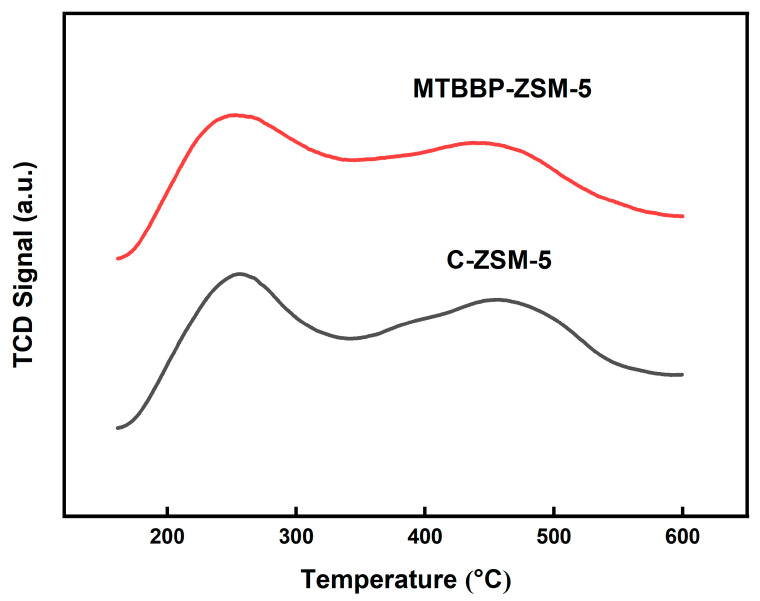
NH_3_-TPD desorption curve of MTBBP-ZSM-5 and C-ZSM-5.

Acidities were investigated by NH_3_-TPD and Py-IR. The semblable NH_3_-TPD curves of C-ZSM-5 and MTBBP-ZSM-5 before Zn impregnation are shown in Figure 6. A low-temperature peak located at ~250 °C and a high-temperature one at 450 °C were distinguished, which can be used to describe the strength and amount of strong or weak acid sites. The quantification analysis, as listed in Table 2, identified that the total acidity amount was 242.8 μmol·g^−1^ for MTBBP-ZSM-5 and 264.8 μmol·g^−1^ for C-ZSM-5.

For the samples after Zn impregnation, significant reductions in strong acid peaks were observable in the corresponding NH_3_-TPD profiles (Figure S3). However, slight increases in the total acidities can be seen in Table 2, indicating the Zn engagements could transform several strong acid sites into weak sites. It was also noteworthy that the acid properties of the two samples were comparable to each other before and after Zn impregnation, as can be inferred from the area and peak locations (Figure 6 and Figure S3).

**Figure 7:**
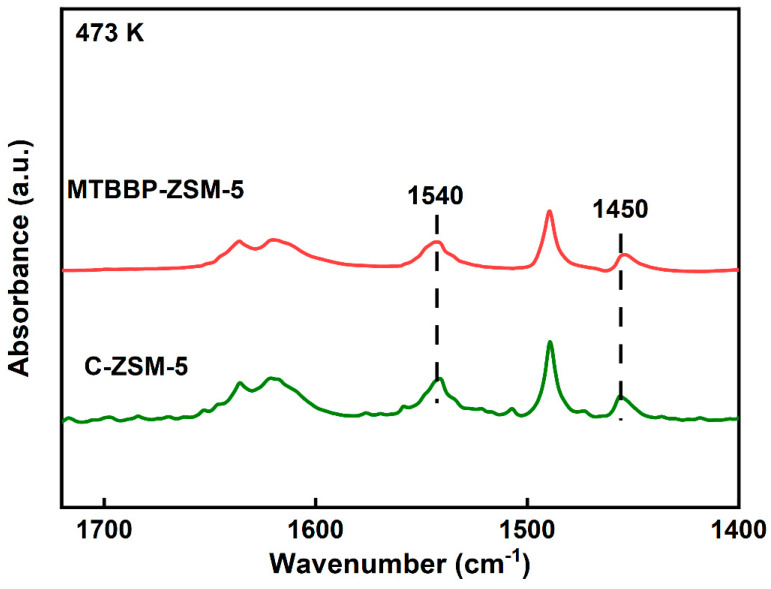
Py−IR spectra of MTBBP−ZSM-5 and C−ZSM−5.

The NH_3_-TPD curves only provide information on total acidity and strength, which cannot be used to differentiate the types of acid sites [44]. As a supplement, pyridine adsorption infrared spectra (Py-IR) were used to investigate Brønsted acid sites (BAS) and Lewis acid sites (LAS), as shown in Figure 7 and Figure S4 and Table 3. The peak at 1540 cm^−1^ was ascribed to the N-H bond in the protonated Pyridium cation absorbed on BAS [37,45], while the peak at 1450 cm^−1^ corresponded to the C-N bond vibration in the pyridine molecule adsorbed on the LAS [46]. As shown in Table 3 and Figure 7, the BAS concentration of MTBBP-ZSM-5 was 264.6 μmol·g^−1^, which is slightly lower than the value of C-ZSM-5 (297.2 μmol·g^−1^). After Zn impregnation, decrements in BAS and increments in LAS could be observed resulting from the replacement of protons by Zn species and the generation of Zn-related LAS [47].

Based on the above analysis, it was obvious that the addition of disinfectants (MTBBP) changed the morphology of the obtained zeolites, but their micro-structures were well retained. Mesopores and macropores could be introduced with the help of disinfectants, in contrast to conventional ZSM-5, which is a microporous material. Among all the disinfectants used, MTBBP was the most excellent one, which resulted in rice-crust-like surfaces possessing a hierarchical structure, and the acid sites remained almost intact.

### 3.2. Crystallization Process Analysis

**Figure 8:**
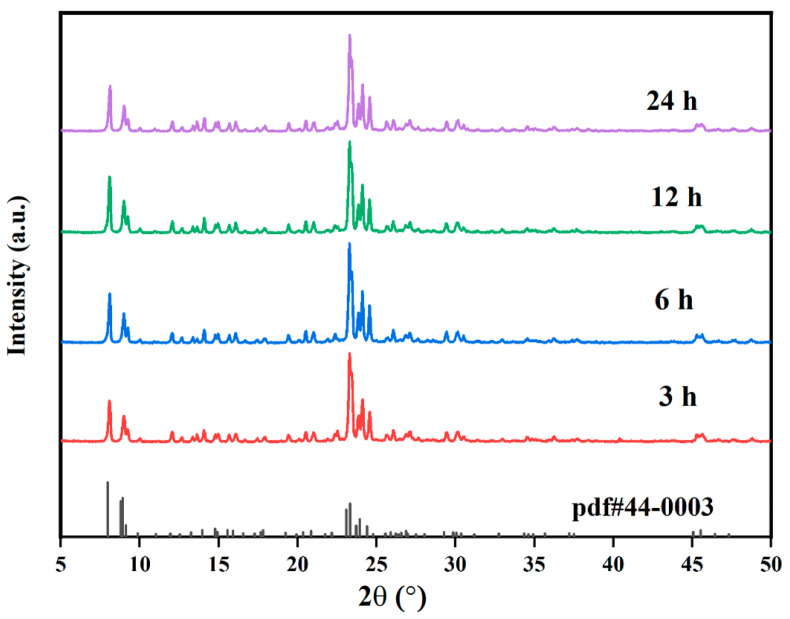
Time-dependent XRD patterns of MTBBP at various crystallization periods.

**Figure 9:**
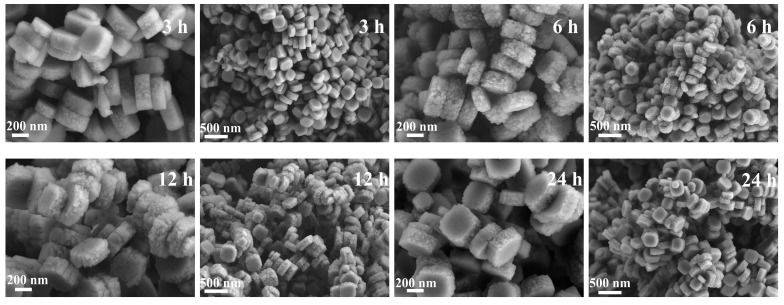
Time-dependent SEM micrographs of MTBBP at various crystallization periods.

MTBBP-ZSM-5 was selected to investigate the effect of disinfectants on the zeolite growth process. The crystallization was periodically interrupted to extract the solid intermediates. These intermediates were then characterized by XRD and SEM. These findings are presented in Figure 8, Figure 9, Figures S5 and S6. A plausible crystallization mechanism of MTBBP-ZSM-5 was proposed based on the above findings.

Figure S6 presents the SEM images of the hydrothermal products at 1 h. This sample consisted of amorphous particles, which are interpreted as irregular aggregations of nano-sized worm-like particles. This observation is further supported by the XRD patterns in Figure S5, in which only the amorphous phase can be seen. When the hydrothermal treatment was prolonged to 3 h, amorphous particles and rough hexagonal slates co-exist in the products, as shown in Figure 9. The XRD results at 3 h, as portrayed in Figure 8, also proved the successful formation of ZSM-5 zeolite. Thereafter, the intensities of XRD signals were saturated, and no significant changes could be observed in the corresponding SEM images. This finding aligned with previous studies that have also observed a rapid amorphous-to-crystalline transformation during the early stages of ZSM-5 crystallization [48].

Considering that there might be a competitive relationship between the Na^+^ cation and the disinfectants, samples with different Na/Al ratios were synthesized to examine their respective roles. Figure S7 displays the SEM images of samples obtained at Na/Al = 0.5, and the related XRD patterns are presented in Figure S8. Unlike the previous observations in Figure S2, abundant particles with rather small sizes could be distinguished in all samples, suggesting the existence of unconverted precursors, while only several incompletely crystallized particles could be found in MTBBP-ZSM-5 and DDAC-ZSM-5. This result indicated that Na^+^ was necessary for the production of ZSM-5 zeolite, possibly because the low charge densities of TPA^+^ and MTBBP could not balance the framework charge. Actually, nothing other than amorphous worm-like particles could be obtained in the totally Na^+^-free systems (Figure S9), and a higher Na^+^ ratio (Na^+^/Si = 0.2) yielded conventional coffin-shaped crystals like C-ZSM-5. In addition, the phosphorus contents of the mother liquid before and after hydrothermal treatment were determined as 0.45 wt.% and 0.47 wt.%, respectively, both of which were very close to the theoretical value of 0.5%. Therefore, it could be concluded that MTBBP was essentially a kind of zeolite growth modifier that cannot enter zeolite frameworks, and it was feasible for cyclic utilization after a simple filtration process.

Taking everything into consideration, MTBBP-ZSM-5 was a result of the competition-coordination relationships between MTBBP and sodium cations. MTBBP should interact with zeolite precursors via Coulomb force as the aluminosilicates were negatively charged [49] and MTBBP has a positive charge. Accordingly, MTBBP would be adsorbed on the precursors during crystal growth, yet its large molecular size rendered it incapable as a template, and it could only fulfill the role of a growth modifier. At the same time, the large size of MTBBP led to a low charge density, implying its interaction with the aluminosilicates is weak. Thus, in a Na^+^-rich environment, the attached MTBBP was possibly substituted by Na^+^ considering Na^+^ owns higher charge density as well as stronger interactions with aluminosilicates, which finally generated traditional smooth hexagonal particles. However, the negative charges of the ZSM-5 zeolite cannot be properly compensated solely by TPA^+^, and Na^+^ must be engaged in the system. Resultantly, the relative contents of Na^+^ and MTBBP were decisive for the successful formation of the “rice crust” morphology, as a low Na^+^ content will lead to failed crystallization, while a high one will eliminate the effects of MTBBP.

### 3.3. Catalytic Performance Evaluation

**Figure 10:**
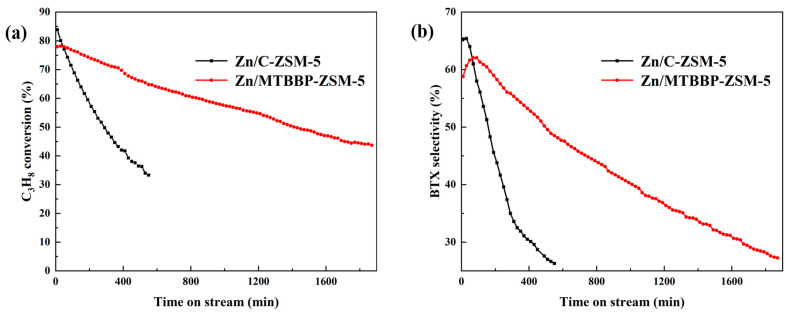
The conversion of propane aromatization with time on stream (TOS) over different catalysts (**a**); BTX selectivity with time on stream (TOS) over different catalysts (**b**).

**Figure 11:**
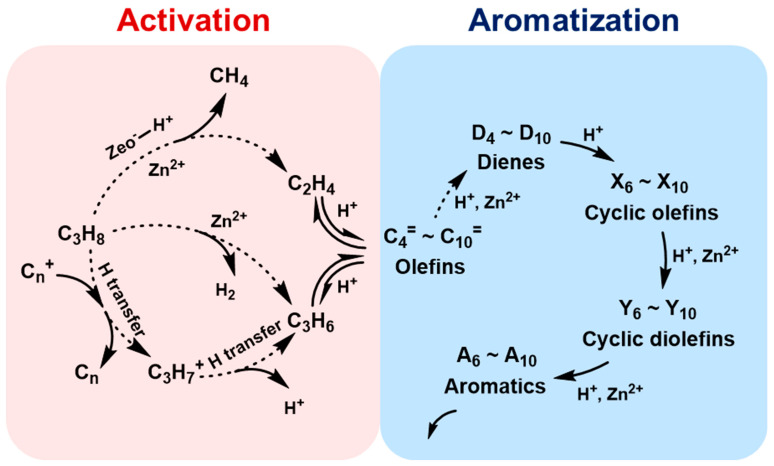
The reaction paths of propane on Zn/ZSM−5 molecular sieve (dashed lines represent slower reactions).

The as-prepared MTBBP-ZSM-5 and C-ZSM-5 were loaded with Zn to afford Zn/MTBBP-ZSM-5 and Zn/C-ZSM-5, which were used as catalysts in propane aromatization. The catalytic performances are displayed in Table 4 and Figure 10. The catalyst was deemed deactivated when propane conversion was lower than 50%, and the lifetimes were determined as 290 min for Zn/C-ZSM-5 and 1430 min for Zn/MTBBP-ZSM-5. Before describing the reaction results, it was useful to elucidate the mechanism again (Figure 11). This reaction began with the activation of propane. Propane can be turned into propene via hydride transfer or dehydrogenation, and propane can also be transformed into methane and ethene via α-scission [50,51,52]. Propene and ethene are rather reactive and undergo oligomerization to afford C_4_~C_10_ olefins, then the olefins are further converted into dienes. Once dienes are formed, they can yield aromatics through cyclization and aromatization. When Zn-modified ZSM-5 was used as a catalyst, dehydrogenation processes mainly took place over Zn-related active sites; meanwhile, other reactions like cyclization and oligomerization depended on the acid sites.

Propane conversions were plotted against time on stream, as shown in Figure 10. The initial propane conversion over Zn/C-ZSM-5 was 83.8% (10 min), which was higher than the conversion over Zn/MTBBP-ZSM-5 at 77.9% (10 min). However, the conversion dropped rapidly for Zn/C-ZSM-5 to 48.9% at 300 min and further to 33.6% at 550 min. In contrast, the propane conversion over Zn/MTBBP-ZSM-5 declined only from 77.9% to 71.7% in the same period and reached 64.8% at 550 min. The total turnover number (TON) for the two catalysts was calculated to be 0.98 mol_C3H8_·g_cat_^−1^ and 0.25 mol_C3H8_·g_cat_^−1^ for Zn/MTBBP-ZSM-5 and Zn/C-ZSM-5, respectively. The TON of Zn/MTBBP-ZSM-5 was approximately fourfold that of Zn/C-ZSM-5, which highlighted the advantage of Zn/MTBBP-ZSM-5 in terms of catalytic stabilities. Combined with previous characterizations, the higher initial propane conversions over Zn/C-ZSM-5 could be attributed to its stronger acidities, as concluded from the NH_3_-TPD and Py-IR results. On the other hand, the better reaction stability of Zn/MTBBP-ZSM-5 should arise from its hierarchical structure, which shortened the residence time and alleviated coke formation.

The trend in BTX selectivity changed similarly to the conversions. At 10 min, the total BTX selectivity of Zn/C-ZSM-5 was 65.3%, which was higher than 58.8% for the counterpart. Nevertheless, BTX selectivity for Zn/C-ZSM-5 suffered from a steep decline of ~30% within the first 300 min, while the drop was only ~3% for Zn/MTBBP-ZSM-5. As mentioned above, the formation of aromatics depended on both the acidities and Zn species. Even though Zn/C-ZSM-5 manifested a higher BTX selectivity at the onset of the reaction, the sluggish diffusion and strong acidities induced more cokes, which blocked the channels and covered the active sites. Hence, the BTX selectivity over Zn/C-ZSM-5 dropped significantly relative to Zn/MTBBP-ZSM-5. Table 4 compares the specific product distributions. In addition to BTX, the other products included methane, ethene, ethane, and propene. C4~C5 species were not detected as they are more reactive and can be cracked or aromatized once formed [53]. For the product distributions at identical TOSs, the conversion of Zn/MTBBP-ZSM-5 was higher than that of Zn/C-ZSM-5 (71.7% vs. 48.9%), which originated from the lower deactivation rate of Zn/MTBBP-ZSM-5. Also at this time, the methane selectivity over Zn/C-ZSM-5 (17.4%) was obviously higher than that of Zn/MTBBP-ZSM-5 (10.8%), indicating enhanced α-scission as a result of pore blockage and retarded diffusion [54]. Meanwhile, the total aromatics selectivity over Zn/C-ZSM-5 was only 34.3% relative to 52.9% for the counterpart, which could be attributed to the coverage of active centers by cokes. The reaction results at comparable propane conversions are also juxtaposed in Table 4, where Zn/C-ZSM-5 manifested a propane conversion of 71.5% and a total aromatic selectivity of 55.6% at 90 min, very close to the date of Zn/MTBBP-ZSM-5 at 300 min. Despite the similar performance, the main difference was the ethane/ethene ratio: for Zn/MTBBP-ZSM-5, the value was 3.7, and for Zn/C-ZSM-5, the value was 7.9, implying the hydride transfer reaction was more severe in Zn/C-ZSM-5 [55]. In terms of the similar conversion, which denotes an equivalent number of available active sites, exacerbated hydride transfer should arise from a sluggish diffusion for Zn/C-ZSM-5 because of the absence of secondary pores or channels. Notably, the hydride transfer reaction is believed to be the primary motivation for the emergence of heavy aromatics that are regarded as coke precursors [56]. Hence, poor diffusion was the main reason for the weak stability of Zn/C-ZSM-5, which resulted in a higher rate for the formation of cokes because of the prolonged residence time and the intensified hydride transfer reaction.

**Figure 12:**
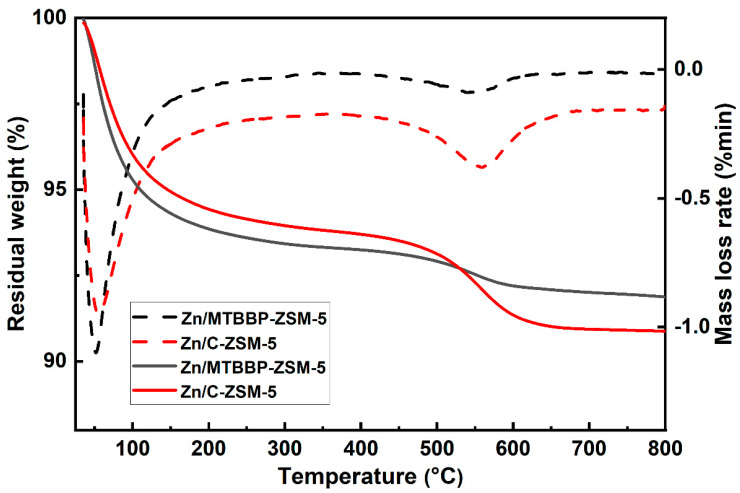
TG and DTG curves of different spent catalysts.

TG was adopted to further investigate the amount of carbonaceous deposits in the spent catalysts (at 50% conversion), and the results are shown in Figure 12. As can be seen in the DTG curves, the weight loss before 300 °C was due to dehydration, and the weight loss between 400 °C and 650 °C was caused by the oxidative combustion of carbon deposits. The TG curves in Figure 12 evidenced a weight loss of 1.2% for spent Zn/MTBBP-ZSM-5 and a weight loss of 2.7% for spent Zn/C-ZSM-5. The average coking rate corresponding to Zn/MTBBP-ZSM-5 and Zn/C-ZSM-5 was calculated to be 8 × 10^−4^ g_coke_/g_cat_ and 9 × 10^−3^ g_coke_/g_cat_, respectively. Obviously, the hierarchical structure of Zn/MTBBPT-ZSM-5 could accelerate diffusion and suppress hydride transfer, which effectively reduced carbon deposits and protected the active sites. Therefore, the stability of Zn/MTBBP-ZSM-5 was much better than Zn/C-ZSM-5 [57].

## 4. Conclusions

In this research, a series of quaternary ammonium or phosphonium salts were applied as zeolite growth modifiers in the one-step hydrothermal synthesis of hierarchical ZSM-5 zeolite. Compared with the other modifiers, the use of methyltriphenylphosphonium bromide (MTBBP) could yield nano-sized hierarchical ZSM-5 with a “rice crust” morphology feature. It was confirmed that the addition of MTBBP led to aligned tiny particle aggregations without discernable adverse effects on the microstructures and acidities, but abundant meso- and marco- pores were created. In addition, the related crystallization process was also discussed. The generation of the special morphology was believed to be a result of the coordination and competition between MTBBP and Na cations. The catalytic performance of the as-synthesized hierarchical ZSM-5 loaded with Zn was tested in propane aromatization, which possessed enhanced catalytic properties and a prolonged lifetime. Lifetime and enhanced total turnover numbers were nearly fivefold longer (1430 min vs. 290 min) and fourfold larger (0.98 vs. 0.25) than traditional ZSM-5. The superior catalytic performance was ascribed to the attenuated hydride transfer reaction and slow coking rate. Introducing mesopores can help alleviate diffusion limitations in ZSM-5, yet achieving precise control over the morphology and pore channels of multi-level pore ZSM-5 molecular sieves remains a challenging task in the academic field. The findings in this research provide a new strategy for synthesizing hierarchical ZSM-5 with surface modification that is less time-consuming and energy-consuming than the two-step treatment, and it is believed that this synthesis strategy can be extended to other porous materials in the near future.

## Figures and Tables

**Table 1:** Textural properties of the synthesized samples derived from N_2_ adsorption–desorption isotherms.

Sample	S_BET_ (m^2^·g^−1^)	S_ext_ (m^2^·g^−1^)	S_micro_ (m^2^·g^−1^)	V_total_ (cm^3^·g^−1^)	V_micro_ (cm^3^·g^−1^)	V_meso_ (cm^3^·g^−1^)
MTBBP-ZSM-5	373	195	178	0.40	0.11	0.29
C-ZSM-5	322	141	181	0.23	0.11	0.12

**Table 2:** Acid properties of the synthesized samples by NH_3_-TPD.

Sample	Total Acidity Amount (μmol·g^−1^)
MTBBP-ZSM-5 C-ZSM-5 Zn/MTBBP-ZSM-5	242.8 264.8 267.5
Zn/C-ZSM-5	286.8

**Table 3:** Acid properties of the synthesized samples by Py-IR.

Sample	Brønsted Acid Amount (μmol·g^−1^)	Lewis Acid Amount (μmol·g^−1^)
MTBBP-ZSM-5 C-ZSM-5 Zn/MTBBP-ZSM-5	264.6 297.2 91.5	70.2 76.4 368.8
Zn/C-ZSM-5	107.6	392.5

**Table 4:** Product distributions of Zn/MTBBP-ZSM-5 and Zn/C-ZSM-5.

Catalyst	Conversion (%)	Selectivity (%)							
		CH_4_	C_2_H_4_	C_2_H_6_	C_3_H_6_	C_6_H_6_	C_7_H_8_	C_8_H_10_	Total Aromatics
**Zn/MTBBP-ZSM-5** **(300 min)**	71.7	10.8	6.8	25.4	4.1	23.5	21.6	7.8	52.9
Zn/C-ZSM-5 (300 min)	48.9	17.4	9.5	33.6	5.2	14.1	13.3	6.9	34.3
Zn/C-ZSM-5 (90 min)	71.5	11.6	3.3	26.0	3.5	24.1	23.0	8.5	55.6

## Data Availability

Data are available upon request.

## Supplementary Materials

The following supporting information can be downloaded at: https://www.mdpi.com/article/10.3390/nano14090802/s1, Figure S1. XRD patterns of TBTCP-ZSM-5, DDAC-ZSM-5, and DDBAC-ZSM-5; Figure S2. SEM micrographs of TBTCP-ZSM-5 (a), DDAC-ZSM-5 (b), and DDBAC-ZSM-5 (c); Figure S3. NH_3_-TPD desorption curves of Zn/MTBBP-ZSM-5 and Zn/C-ZSM-5; Figure S4. Py-IR spectra of Zn/MTBBP-ZSM-5 and Zn/C-ZSM-5; Figure S5. XRD patterns of synthesized MTBBP-ZSM-5 within 1 h; Figure S6: SEM micrographs of synthesized MTBBP-ZSM-5 within 1 h; Figure S7. SEM micrographs of MTBBP-ZSM-5 (a), TBTCP-ZSM-5 (b), DDAC-ZSM-5 (c), and DDBAC-ZSM-5 (d) synthesized with Na/Al = 0.5; Figure S8. XRD patterns of synthesized samples with Na/Al = 0.5; Figure S9. SEM micrograph of MTBBP-ZSM-5 synthesized with Na/Al = 0; Table S1. Textural properties of the synthesized samples derived from N2 adsorption-desorption isotherms.
