# Supplementary material for: Disinfectant-Assisted Preparation of Hierarchical ZSM-5 Zeolite with Excellent Catalytic Stabilities in Propane Aromatization
Source: Nanomaterials (Basel). 2024 May 5;14(9):802. doi: 10.3390/nano14090802 (PMC11085285; doi:10.3390/nano14090802)
Supplement: Supplementary file 1 [file nanomaterials-14-00802-s001.zip › nanomaterials-2971944-supplementary.pdf]

Supplementary Material

Disinfectant-Assisted Preparation of Hierarchical ZSM-5 Zeolite with Excellent Catalytic Stabilities in Propane Aromatization

Peng Zhang [1], Jianguo Zhuang [1], Jisheng Yu [1], Yingjie Guan [1], Xuedong Zhu [1,*] and Fan Yang [2,*]

[1] Engineering Research Center of Large-Scale Reactor Engineering and Technology, East China University of Science & Technology, Ministry of Education, Shanghai 200237, China; zhangpengxxx123@163.com (P.Z.)

[2] State Key Laboratory of Green Chemical Engineering and Industrial Catalysis, Sinopec Shanghai Research Institute of Petrochemical Technology Co., Ltd., Shanghai 201208, China

* Correspondence: xdzhu@ecust.edu.cn (X.Z.); yangfan.sshy@sinopec.com (F.Y.)

List of contents

Fig. S1. XRD patterns of TBTCP-ZSM-5, DDAC-ZSM-5 and DDBAC-ZSM-5.

Fig. S2. SEM micrographs of TBTCP-ZSM-5(a), DDAC-ZSM-5(b) and DDBAC-ZSM-5(c).

Fig. S3. NH_3-TPD desorption curves of Zn/MTBBP-ZSM-5 and Zn/C-ZSM-5.

Fig. S4. Py-IR spectra of Zn/MTBBP-ZSM-5 and Zn/C-ZSM-5.

Fig. S5. XRD patterns of synthesized MTBBP-ZSM-5 within 1 hour.

Fig. S6. SEM micrographs of synthesized MTBBP-ZSM-5 within 1 hour.

Fig. S7. SEM micrographs of MTBBP-ZSM-5(a), TBTCP-ZSM-5(b), DDAC-ZSM-5(c) and DDBAC-ZSM-5(d) synthesized with Na/Al = 0.5.

Fig. S8. XRD patterns of synthesized samples with Na/Al = 0.5.

Fig. S9. SEM micrograph of MTBBP-ZSM-5 synthesized with Na/Al = 0.

Table S1. Textural properties of the synthesized samples derived from N_2 adsorption-desorption isotherms.

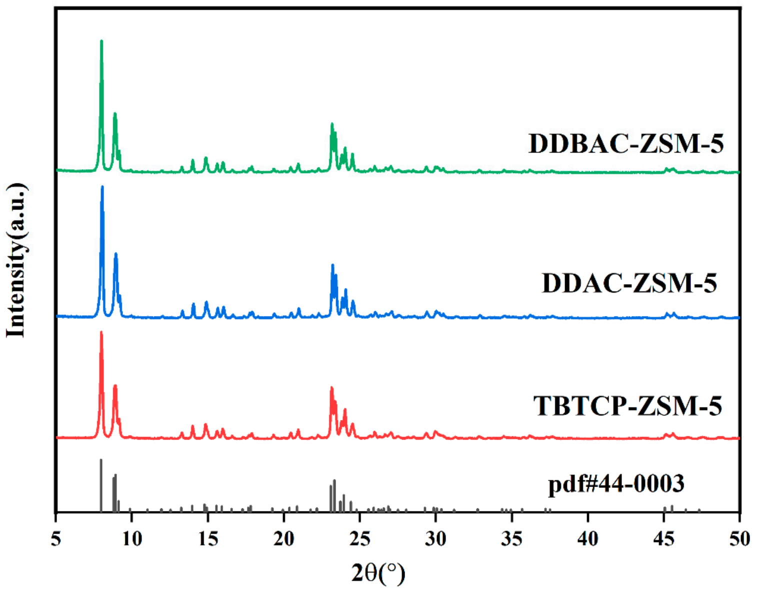

Fig. S1. XRD patterns of TBTCP-ZSM-5, DDAC-ZSM-5 and DDBAC-ZSM-5.

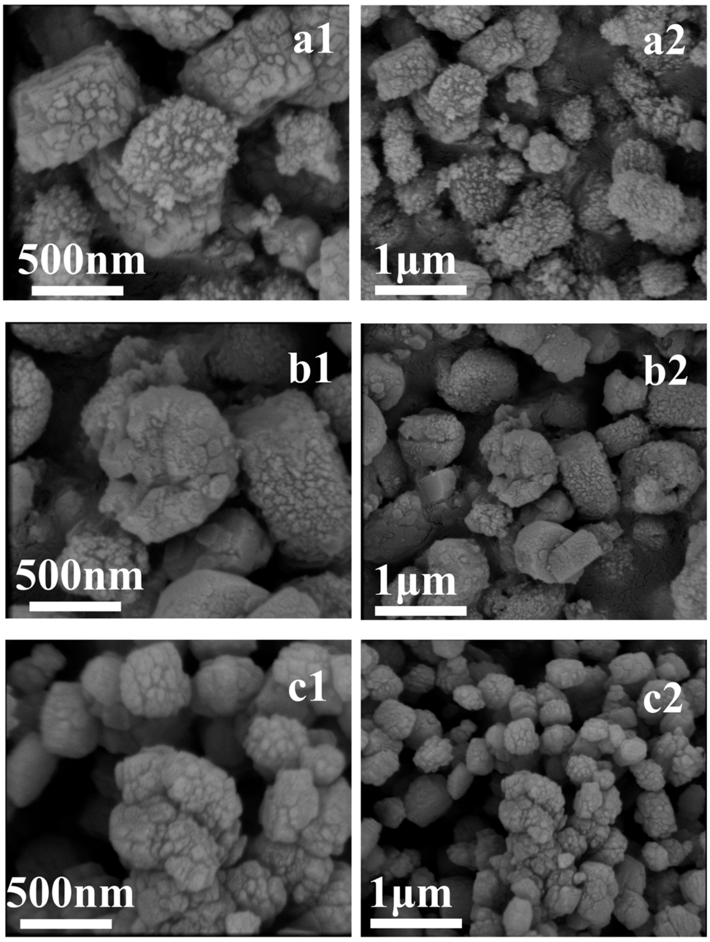

Fig. S2. SEM micrographs of TBTCP-ZSM-5(a), DDAC-ZSM-5(b) and DDBAC-ZSM-5(c).

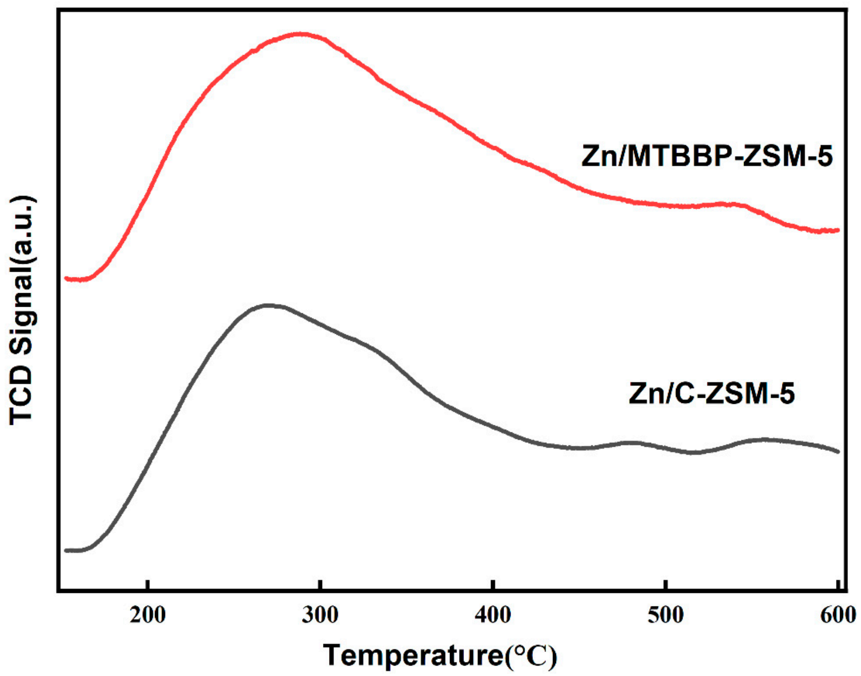

Fig. S3. NH_3-TPD desorption curves of Zn/MTBBP-ZSM-5 and Zn/C-ZSM-5.

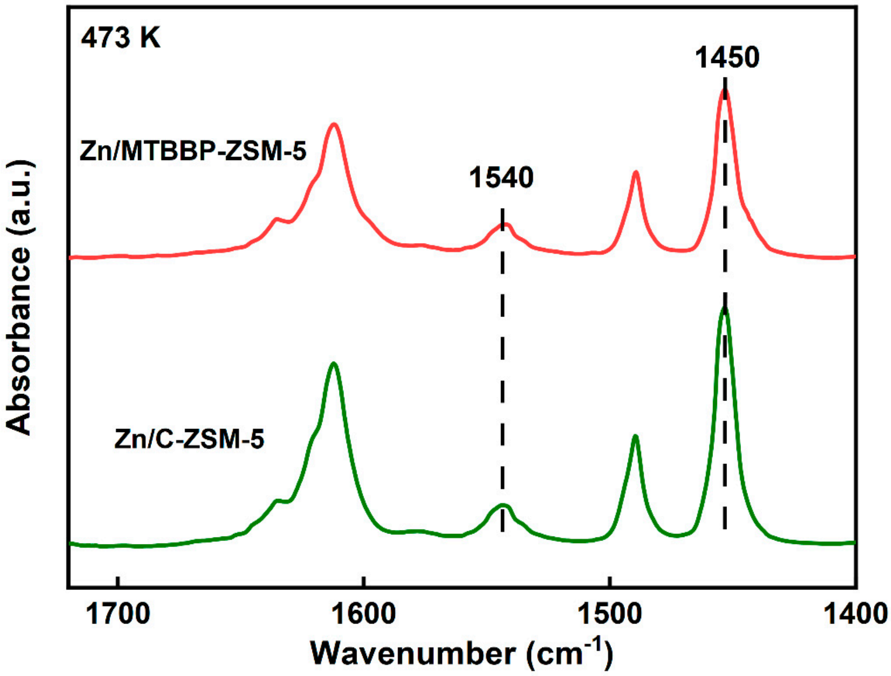

Fig. S4. Py-IR spectra of Zn/MTBBP-ZSM-5 and Zn/C-ZSM-5.

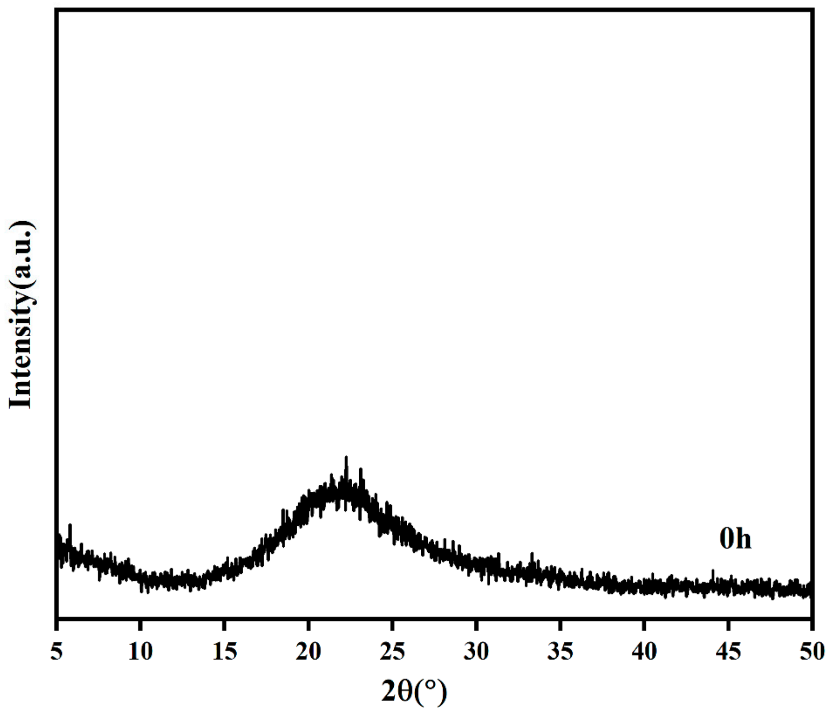

Fig. S5. XRD patterns of synthesized MTBBP-ZSM-5 within 1 hour.

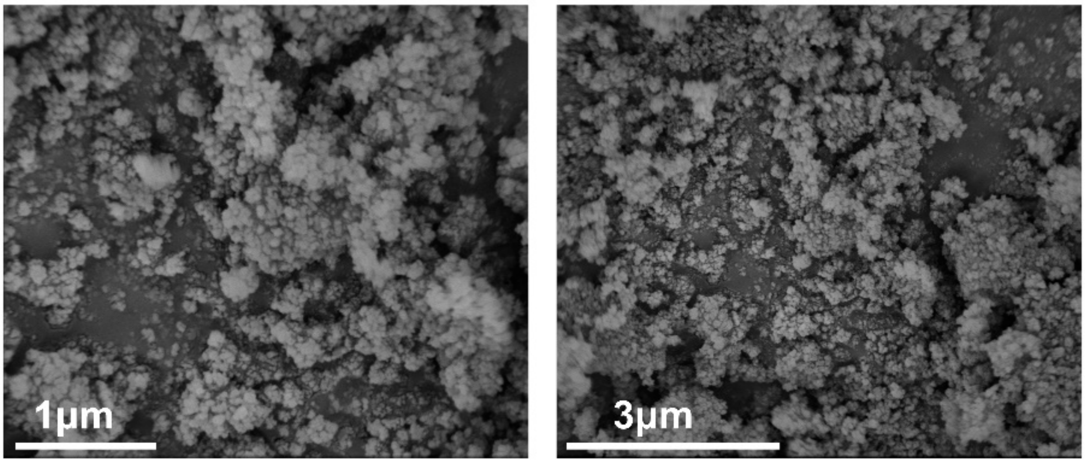

Fig. S6. SEM micrographs of synthesized MTBBP-ZSM-5 within 1 hour.

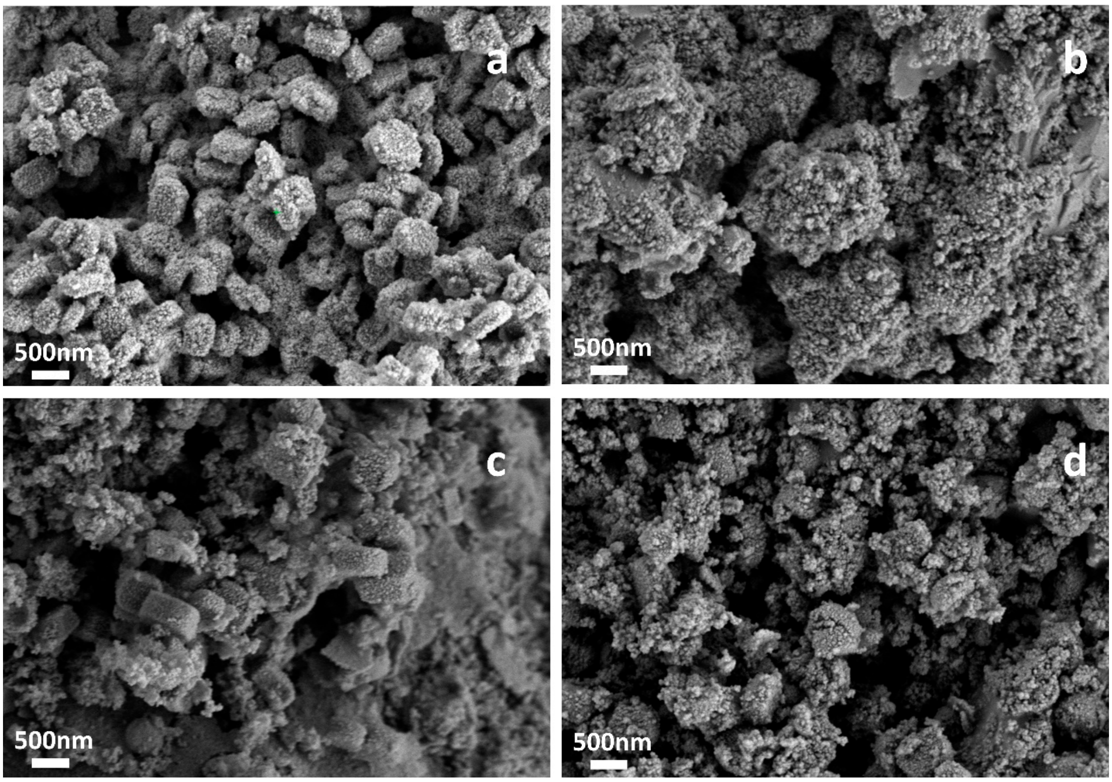

Fig. S7. SEM micrographs of MTBBP-ZSM-5(a), TBTCP-ZSM-5(b), DDAC-ZSM-5(c) and DDBAC-ZSM-5(d) synthesized with Na/Al = 0.5.

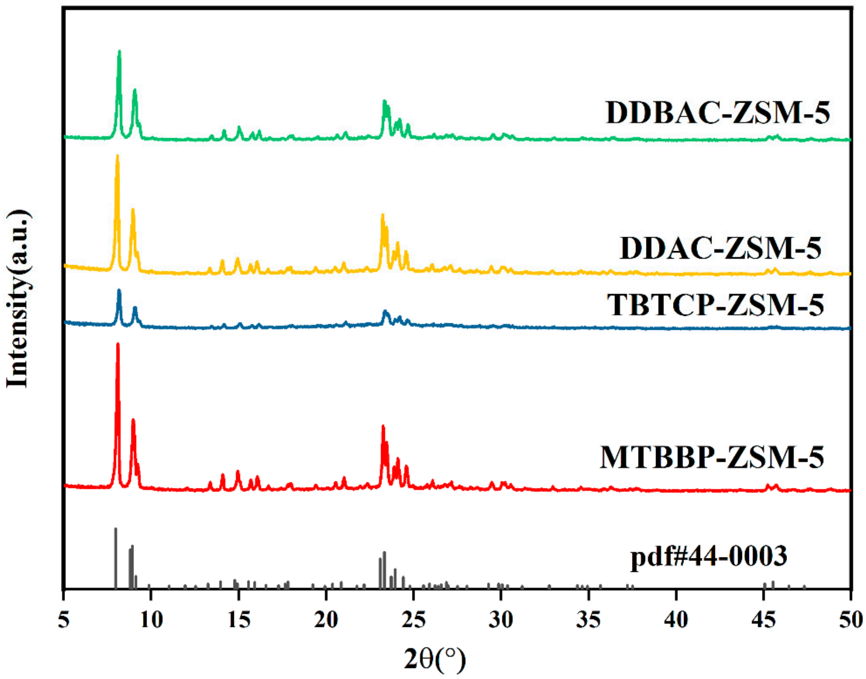

Fig. S8. XRD patterns of synthesized samples with Na/Al = 0.5.

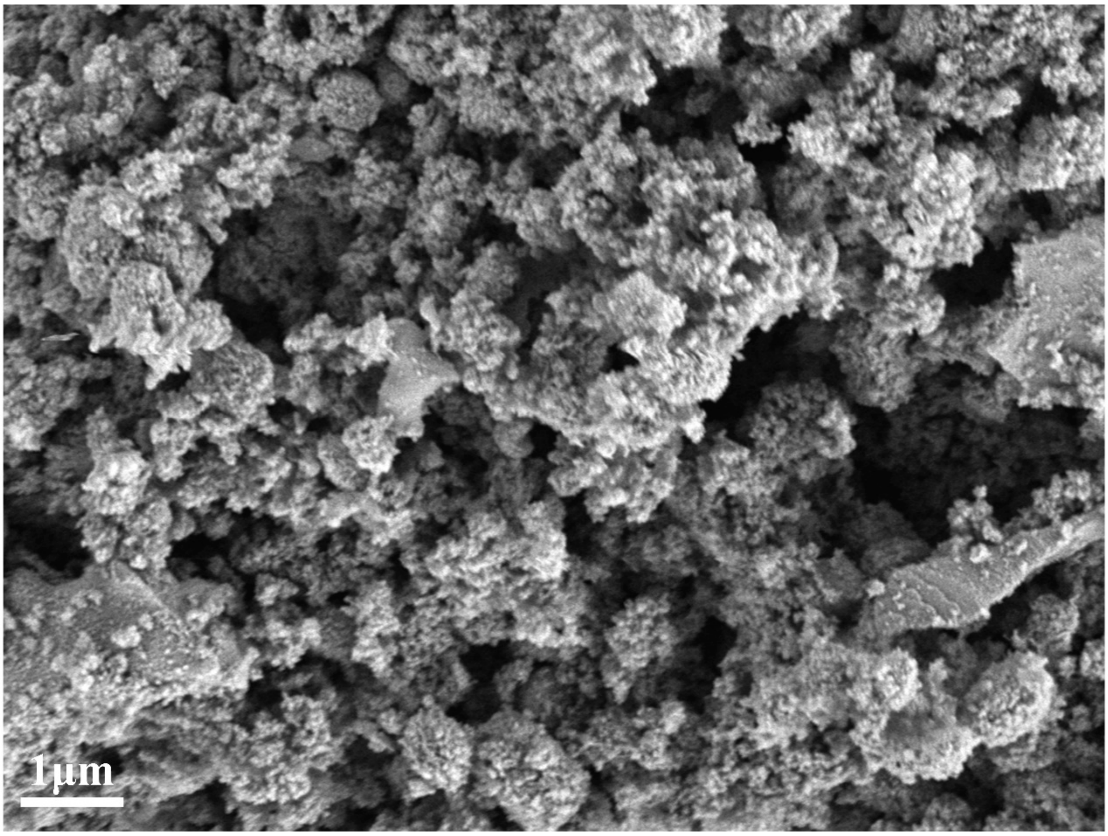

Fig. S9. SEM micrograph of MTBBP-ZSM-5 synthesized with Na/Al = 0.

Table S1. Textural properties of the synthesized samples derived from N_2 adsorption-desorption isotherms.

Sample	S_{BET} ($m^2 \cdot g^{-1}$)	S_{ext} ($m^2 \cdot g^{-1}$)	S_{micro} ($m^2 \cdot g^{-1}$)	V_{total} ($cm^3 \cdot g^{-1}$)	V_{micro} ($cm^3 \cdot g^{-1}$)	V_{meso} ($cm^3 \cdot g^{-1}$)
MTBBP	373	195	178	0.40	0.11	0.29
TBTCP-ZSM-5	313	137	176	0.22	0.13	0.09
DDAC-ZSM-5	317	144	173	0.22	0.09	0.13
DDBAC-ZSM-5	326	141	185	0.21	0.10	0.11
